# Clinical review of humic acid as an antiviral: Leadup to translational applications in clinical humeomics

**DOI:** 10.3389/fphar.2022.1018904

**Published:** 2023-01-04

**Authors:** David C. Socol

**Affiliations:** ^1^ Advanced Humeomics LLC, Beverly Hills, CA, United States; ^2^ SocolMD, Beverly Hills, CA, United States

**Keywords:** Humic acid (HA), fulvic acid, shilajit, humic substances, carboxylated polyanions, antiviral, extracellular and intracellular mechanisms of action

## Abstract

This clinical review presents what is known about the antiviral features of humic substances (HS) to the benefit of the clinical healthcare provider using available data in humeomics, the study of the soil humeome. It provides the reader with a working framework of historical studies and includes clinically relevant data with the goal of providing a broad appreciation of the antiviral potential of humic substances while also preparing for a translational leap into the clinical application of humic acid.

## Introduction

Humic substances include a variety of chromogenic, or pigmented, organic molecules that are primarily distributed in soils, rivers, oceans, and iterations of coal (Jung et al., 2021). They are also found in small quantities in Chaga (*Inonotus obliquus)*, though this iteration contains almost no nitrogen in contrast to primary reservoirs of humic substances (Shashkina et al., 2006).

The soil humeome has the greatest abundance of humic substances and is fundamental to plant growth, carbon storage, and the management of environmental contaminants (Orsi, 2014). It, as well as other sources of humic substances, originated in the vast fauna which carpeted the biosphere 50–280 million years ago, between the Cenozoic and Paleozoic eras. Though the principal molecules of the soil humeome, namely humic acid, fulvic acid, and humin, are highly conserved across the environment, it is clinically valuable to regard humic acid as a molecular genus rather than a well-defined molecule with a static molecular formula and three-dimensional conformation. Differences in local fauna, micro-environments, and a time factor yield “variations on a theme” at the molecular level (Stevenson, 1994), which has a bearing on chemistry and ultimately clinical potential. Despite their common molecular superstructure, the highly iterative origin of the humic acids contrasts with conventional standards of pharmacology and our expectations in clinical practice (Murbach et al., 2020).

**FIGURE 1:**
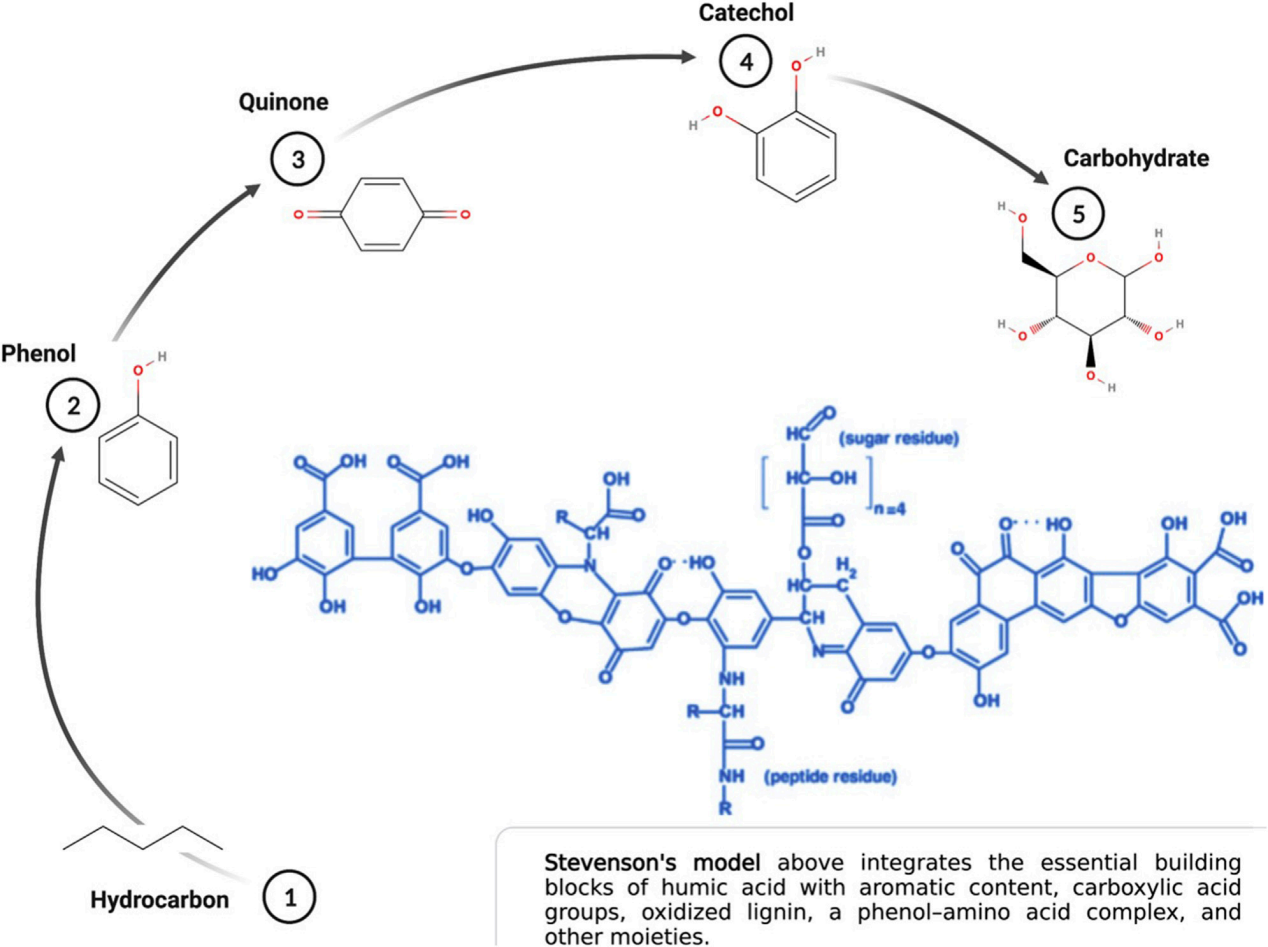
Essential building blocks of humic acid. Created with Biorender.com.

The conceptual model of humic acid has evolved dramatically over the past 20 years in tandem with new technologies that assay the soil humeome. The classical model of humic acid, that of a molecular polymer with an organized and predictable structure, has been superseded by a complex model based on a supramolecular aggregate of smaller molecules derived from the biotic and abiotic degradation of dead plant matter (Piccolo et al., 2018). Though the assembly of the supramolecular aggregate depends on an array of hydrophobic interactions and hydrogen bonds across multiple relatively low molecular mass sub-components, the dimensional behavior of these biomolecular components create unique molecular micro-environments that contribute to humic acid chemistry and the nuance between humic acid molecules. That these biomolecules are also indivisible from the larger humic molecule amends the functional paradigm of what it means to be a humic acid (Sutton and Sposito, 2005). It also makes it that much more difficult to conceptualize the three-dimensional conformation of humic acid given these added layers of complexity (Ghabbour et al., 2001). Regardless of these advancements, the building blocks of humic acid remain well established (Figure 1). The influence of carboxylic, alcoholic, and sulfhydryl groups to the supramolecule’s chemistry, in addition to fatty acids, amino acids and polypeptides, differentiate the functional potential of each humic acid iteration (Sutton and Sposito, 2005; Al-Faiyz, 2013; Orsi, 2014; Bondareva and Kudryasheva, 2021).

This complex backstory notwithstanding, the clinical value of the soil humeome was first identified by indigenous cultures living within the Himalayan regions of Bhutan, India, Nepal and Pakistan, in the form of shilajit, or mineral pitch, with a minor contribution emanating from Tibet and China (Meena et al., 2010). Shilajit, or a blend of metallo-humates, including humic and fulvic acids, low and medium molecular weight non-humic organic compounds, and medium and high molecular weight dibenzo-alpha-pyrones-chromoproteins, is still used today as an Ayurvedic medicinal food to mitigate a variety of physical ailments (Ghosal, 2006). Nearly 90-years of scientific research beginning in the 1930s has associated these molecules with anti-inflammatory (Junek et al., 2009), anti-oxidant (Zykova et al., 2018; Bondareva and Kudryasheva, 2021; Kulikova and Perminova, 2021), antiviral (See Table 1), anti-cancer, mycotoxin binding (De Mil et al., 2015) and gut-promoting properties (*via* optimization of the microbiota), though often without distinguishing between the relative contribution of humic acid versus fulvic acid to the net clinical effect (Vetvicka et al., 2010; Kuhnert, 2011; Vetvicka et al., 2013; Winkler and Ghosh, 2018; Lavrik and Ilyitcheva, 2019).

**TABLE 1:** The antiviral spectrum of humic substances.

Virus	Author/Publication
Coronavirus - SARS-Cov-2	Hafez et al. (2020); Hajdrik et al. (2022), Vladimirovna et al. (2021)
Coxsackie virus A9	Klöcking and Sprössig. (1972); Klöcking and Sprössig. (1975)
Cytomegalovirus (human)—CMV	Cagno et al. (2015); Meerbach et al. (2001); NIH. (2002)
Epstein Barr Virus—EBV	NIH. (2002)
Hepatitis B	Pant et al. (2016)
Herpes Simplex Virus Type 1—HSV-1	Cagno et al. (2015); Klöcking et al. (2002); Meerbach et al. (2001); NIH. (2002)
Herpes Simplex Virus Type 2—HSV-2	Cagno et al. (2015); Meerbach et al. (2001); NIH. (2002)
Human Immunodeficiency Virus—HIV-1	Botes et al. (2002); Bruccoleri. (2013); Kornilaeva et al. (2019); Meerbach et al. (2001); Rege et al. (2012); Schneider et al. (1996); van Rensburg et al. (2002); Zhernov. (2018); Zhernov et al. (2017); Zhernov et al. (2021)
Human Immunodeficiency Virus—HIV-2	Meerbach et al. (2001)
Influenza Virus Type A	Lu et al. (2002); NIH. (2002)
Influenza Virus Type B	NIH. (2002)
Pichinde Virus/An 4763	NIH. (2002)
Punta Toro A Virus/Adames	NIH. (2002)
Respiratory Syncytial Virus - RSV	Cagno et al. (2015)
Tick-borne Encephalitis Virus—TBEV	Orlov et al. (2019)
Varicella Zoster Virus—VZV	NIH. (2002)

**FIGURE 2:**
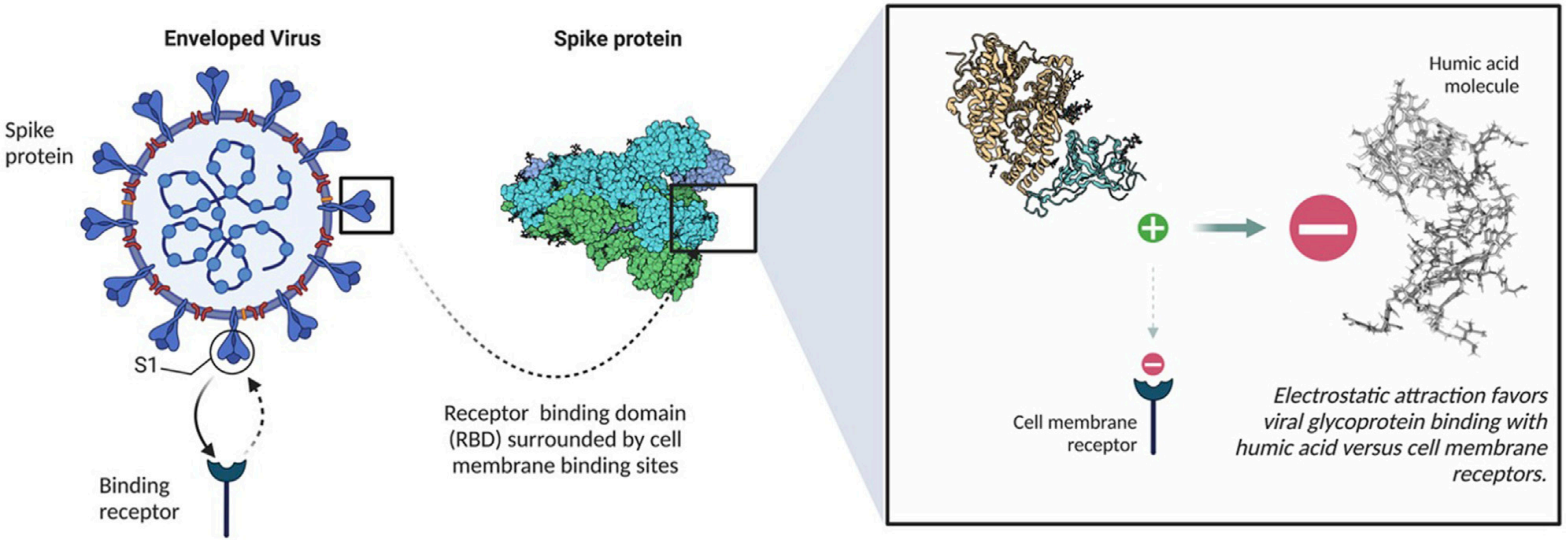
Binding mechanism of humic acid with spike proteins. Created with Biorender.com.

The potential of humic acid as an anti-viral is intrinsic to humic substances as a functional molecular class. These negatively charged polyanionic supramolecules rely on their net negative charge to bind positively charged viral glycoproteins, which ultimately inhibits viral fusion with susceptible cell membrane receptors (Figure 2) *via* a competitive inhibition mechanism. Numerous *in vitro* studies have established the antiviral capacity of humic acid molecules to influence Human Immunodeficiency Virus Types 1 and 2 (HIV-1 and HIV-2), Herpes Simplex Virus Types 1 and 2 (HSV-1 and HSV-2), Epstein Barr Virus (EBV), Varicella Zoster Virus (VZV), Influenza A, Influenza B, Respiratory Syncytial Virus (RSV), human Cytomegalovirus (hCMV), Severe Acute Respiratory Syndrome Coronavirus 2 (SARS-CoV-2), and other virus dynamics through an extracellular mechanism of action. Other studies have illuminated an intracellular antiviral contribution, particularly with HBV, HIV and influenza A (Lu et al., 2002; Pant et al., 2016). Other theoretical mechanisms to antiviral activity, such as cytotoxicity, have been reasonably excluded. Within the humic substance family, humic acid exhibits the greatest antiviral potential relative to its smaller sister molecule, fulvic acid, and shilajit. While multiple studies further conclude that humic substances exhibit no toxic features *in vitro*, others contend that pre-clinical studies are warranted to transition historical research into clinical practice, particularly in consideration of the potential downsides of using humic substances, such as poor bioavailability, heavy metal toxicity, inducement of hypercoagulability, and chelation of essential minerals.

## Humic acid as an antiviral

The SARS-Cov-2 pandemic of 2019 illuminated the veracity of infectious disease, and viral infection in particular, to induce sub-clinical and clinical disease, sabotage patient recovery with a constellation of novel and residual symptoms, disrupt national economies, and impair local, national and geopolitical discourse. The inescapable requirement to reconcile public health needs against a granular, patient-based individual needs analysis requires a diversified toolbox that is considerate of both worlds. The ever-present danger of a SARS-CoV-2 mutation that eludes our antiviral repertoire is unfortunately real, particularly given its rate of antigenic shift and known parallels of drug-resistant viral infections, including influenza vs. adamantane derivatives (Dong et al., 2015), the herpes viradae vs. acyclovir (Pottage and Kessler, 1995; Bacon et al., 2003), and retroviruses vs. azithromycin (Jeeninga et al., 2001). The need to develop supplements and/or adjuncts to vaccines that are indifferent to viral mutation, exhibit prophylactic potential, reduce viral shedding and that modulate the depth and duration of clinical disease bear relevance now and in the future (Neuzil, 2021).

It has long been appreciated that humic substances exhibit antiviral activity, which is based on their molecular feature as carboxylated polyanions with a net negative charge that bind to positively charged viral glycoproteins (Helbig et al., 1997; Klöcking et al., 2002; Jooné et al., 2003). Humic acid is the primary molecule in this molecular family that exhibits antiviral activity, in contrast to other molecules in the HS fraction, such as fulvic acid and shilajit, a related natural product. This being said, the origin of humic acid is also relevant to its antiviral potential. Humic acid sourced from coal exhibits greater antiviral activity than samples from peloid and peat, as well as synthetic iterations of the molecule, such as caffeic acid or chlorogenic acid, versus select viral challenges (Zhernov et al., 2021). Zhernov et alia (2021) further identified the highest antiviral activity in the most hydrophobic and aromatic enriched humic acids. Zhernov et alia’s prior research in 2018 further identified a direct relationship between antiviral activity with the preponderance of CHO (carbohydrate) molecules in humic acid as well as the molecule’s lipophilicty, in addition to an inverse relationship with its density of carboxylic groups and total acidity (Zhernov, 2018). However, it seems that it was Meerbach, as early as 2001, who linked humic acid’s antiviral activity with the presence of carboxylic acid groups (Meerbach et al., 2001).

## *In vitro* antiviral spectrum of activity

Numerous studies have established the activity of natural and synthetic humic substances as antivirals *in vitro* (Table 1). With the exception of Klöcking’s work with Coxsackie virus A9, ECHO-Virus Type 6 and Adenovirus Type 2 in the 1970s (Klöcking and Sprössig, 1972; Klöcking and Sprössig, 1975), these studies largely explored the binding affinity of negatively charged polyanionic substances, or humic substances, with viruses that include positively charged glycoproteins in their viral envelope. Though other studies also reference the binding potential of carboxylated polyanions with non-enveloped viruses, such as Human Papilloma Virus (HPV), the data is limited (Buck et al., 2006) and the relative efficacy still favors enveloped viruses in the absence of molecular enhancements. Others have identified clinical crossover potential of the carboxylated polyanion, poly (styrene-4-sulfonate) against the bacteria *Chlamydia trachomatis* and *Neisseria gonorrhoeae* (Anderson et al., 2000). This being said, relative to other antiviral biomaterials with comparable molecular features, the Selectivity Index (SI), or antiviral activity of natural and synthetic carboxylated polyanions, significantly prefers enveloped viruses over non-enveloped viruses (Terasawa et al., 2020).

## NIH raw data and commentary

In 2002, a contract lab through the National Institutes of Health (NIH) completed a comparative study that examined the relative antiviral activity of humic acid *in vitro* (NIH, 2002). The data from the original manuscript has been reorganized and is presented in Table 3 through six below.

The study performed through the NIH remains a benchmark in the landscape of *in vitro* research on humic substances. Though studies performed since 2002 have taken advantage of technological developments to provide more precise comparative data regarding the antiviral potential of humic substances, the NIH work is notable for its breadth of study, the quality of its data, and the lessons learned based on its conclusions. Subsequent work by others has verified specific conclusions of this study (Meerbach et al., 2001; Cagno et al., 2015; Zhernov et al., 2017; Zhernov, 2018; Zhernov et al., 2021).

For much of its report, the NIH study used IC_50_ and IC_90_ endpoints to quantify the antiviral efficacy of humic acid versus a reference antiviral compound *in vitro*. Table 2A and Table 2B itemize terminology utilized in the NIH report and other like-minded studies which warrant a definition or conceptual framework.

**TABLE 2:** A and B Terminology of terms and viral assays cited in this clinical review.

A	
Term	Refers to
CC_50_	The cytotoxic concentration of an active ingredient that reduces the number of viable cells by 50% in culture relative to a control
EC_50_	The effective concentration of an active ingredient that induces an effect in 50% of the cells in culture relative to control
IC_50_	The inhibitory concentration of an active ingredient that prevented infection in 50% of the cells in culture relative to control
IC_90_	The inhibitory concentration of an active ingredient that prevented infection in 90% of the cells in culture relative to control
TC_50_	The (toxic) concentration of an active ingredient that induces toxic effects in 50% of the cells in culture relative to control
CP_50_	The concentration of an active ingredient that reduces the proliferation of cells in culture by 50% relative to control. CP50 may be used to calibrate mitochondrial integrity in the presence of an active ingredient

B	
Cytopathic Effect, CPE	Identifies morphological changes in cells caused by viral infection, including cell destruction, sub-total destruction, focal degeneration, swelling and clumping, foamy degeneration (vacuolization), cell fusion (syncytium), and the emergence of inclusion bodies. American Society for Microbiology. (2007); CytoSmart. (2020)
Neutral Red Assay, NR	Assesses the ability of viable cells to incorporate and bind neutral red into their lysosomes. Repetto et al. (2008)
Virus Yield, VY	Measures the antiviral activity of a test compound Prichard et al. (1990); Goebel et al. (2016); Creative Diagnostics. (2022)

The results presented in Tables 3 and 4 established several key benchmarks relative to humic acid as an antiviral *in vitro*:

• Relative to the reference compound (acyclovir in Table 3 and ribavirin in Table 4), humic acid demonstrated IC_50_ and IC_90_ results consistent with antiviral activity.
• The IC_50_ and IC_90_ data generated by the studies illuminated the adaptability of the humic acid molecule as a non-specific antiviral.
• The antiviral activity of humic acid was greater for the influenza study (Table 4) than for the herpesviridae study (Table 3) in the aggregate. Within the herpesviridae study, the relative difference between the IC_50_ and IC_90_ findings for HSV-1 and HSV 2 versus VZV and EBV was notable, though meaningful IC_50_ and IC_90_ data was still generated for VZV and EBV.

**TABLE 3:** Effective inhibitory concentration at 50% (IC_50_) and 90% (IC_90_) of humic acid (HA) and acyclovir reference compound with herpesviridae (NIH, 2002).

	IC_50_ mcg/ml		IC_90_ mcg/ml	
	HA	Acyclovir	HA	Acyclovir
HSV1—Herpes Simplex Virus Type I^a^	4.7	1.2–1.6	13.1	7.9
HSV2—Herpes Simplex Virus Type 2^a^	2.5	1.1–1.3	6.7	9.5
VZV—Varicella Zoster Virus^a^	53.5	0.23–0.38	85.8	16.3
EBV—Epstein Barr Virus^b^	>50	1.8–2.4	>50	16.3

^a^ Human foreskin fibroblast cells.

^b^ Daudi cells.

**TABLE 4:** Effective inhibitory concentration at 50% (IC_50_) and 90% (IC_90_) of humic acid (HA) and ribavirin reference compound with influenza virus, Types A and B, in MDCK cells (NIH, 2002).

	IC_50_ mcg/ml						IC_90_ mcg/ml	
	CPE method		NR method		VY method			
	HA	Ribavirin	HA	Ribavirin	HA	Ribavirin	HA	Ribavirin
Type A								
H1N1 New Caledonia/20/99	2.5	0.55	2.5	0.38	3.2	0.32	5	1.4
H3N2 Panama/2007/99	<1	1.3	<1	1.8	0.22	1.9	0.4	1.4
H1N1—NWS/33	1.3	5–6.0	1.3	4.6–6.5	—	—	—	—
H1N1—PR/8/34	14	9	18	12	—	—	—	—
H3N2 Shangdong/09/93	15	1.5–3.2	18	1.7–3.2	—	—	—	—
H3N2 Sydney/05/97	0.35	1	0.55	2	—	—	—	—
Type B								
Beijing/184/93	<1	<1	<1	1.5	0.5	0.5	2.5	1
Harbin/07/94	0.7	0.85	0.65	1.1	—	—	—	—
Hong Kong/5/72	3.2	1.2–1.8	5	1.8–1.8	—	—	—	—

Contrary to the data generated for the Herpesviridae study summarized in Table 3, the influenza virus studies recruited a trifecta of assays to add credence to the dataset (Table 2B). Initial studies to evaluate the ability of natural humates to inhibit the influenza virus’ cytopathic effect (CPE) against cells *in vitro* were followed by neutral red (NR) studies that sought to validate the CPE data by quantifying the intensity of neutral red absorbed by surviving cells. In the final study, natural (and synthetic) humates that were regarded as active by CPE inhibition and confirmed by the NR assay were retested using the CPE method. These samples were then assessed for a reduction of virus yield (VY) relative to a positive control by assaying viral titers in the presence of susceptible cells. Development of CPE in the susceptible cell population was an indication of the presence of infectious virus and an ineffective natural humate.

## Primary extracellular mechanism of action

The antiviral mechanism of humic acid is divided into a dominant extracellular component and a secondary cadre of intracellular mechanisms. In the extracellular framework, negatively charged carboxylated polyanions bind to positively charged viral envelop glycoproteins. Using HIV as a model, humic acid interacts with the positively charged V3 loop of the HIV-1 glycoprotein (gp120) or the glycoprotein 41 (gp41) complex (Zhernov et al., 2017). With SARS-CoV-2, it is theorized that humic acid binds to the positively charged M-glycoprotein of the viral envelop.

Historical studies aimed at deducing the antiviral mechanism of humic substances used assays for cytotoxicity *in vitro* to provide back door insight regarding humic acid’s mechanism(s) of action. In the absence of cytotoxicity, other rationales to explain the antiviral findings *in vitro* would advance to the forefront. Using a combination of toxicity and cell proliferation assays, the NIH’s 2002 study established that humic acid was not cytotoxic at levels at least as high as 100mcg/mL across a variety of cell lines (Table 5). Cagno et alia’s 2015 time-of-addition studies to demonstrate shilajit’s dose-dependent inhibitory activity against HSV-1, HSV-2, hCMV and RSV yielded that shilajit had no influence on cell viability at concentrations as high as 1500 mcg/mL (Cagno et al., 2015). This conclusion further narrowed the antiviral mechanism to something other than cytotoxicity. In 2017, Zhernov et alia similarly concluded that all humic pagination exhibited low cytotoxicity; however, their study design did not reach the CC_50_ for the humic polyanion studied. As a result, the CC_50_ for humic acid was estimated based on the largest concentration tested as > 200 mcg/mL (Zhernov et al., 2017). Zhernov et alia’s subsequent finding in 2021 that mitochondrial activity of cells *in vitro* remained greater than 80% in the presence of humic acid and shilajit samples at a concentration of 1000 mg/L reiterated his prior finding as well as the data published in the NIH study and provides further proof of concept that the mechanism driving the antiviral activity of humic acid and shilajit does not rely on cytotoxicity (NIH, 2002; Zhernov et al., 2021).

**TABLE 5:** Cytotoxicity assays: Humic acid with indicated cell lines (NIH, 2002).

Cell line	Toxic concentration at 50% (TC_50_), mcg/ml	Cell proliferation inhibition concentration at 50% (CP_50_), mcg/ml
African green monkey kidney cells	>100	No data
Human foreskin fibroblast cells	>100	88.4
Madin Darby canine kidney cells	>100	—
Adult rhesus monkey kidney cells	>100	—
	Neutral Red Assay: >1000	
	Visual Assay: >1000	
Daudi Cells (Burkitt’s lymphoma derived cells)	No data	> 50

Ultimately, the 2002 NIH study established the primary mechanism of action of humic polyanions using time-of-addition methodology (Table 6). Studies performed by Meerbach et al. (2001); Cagno et al. (2015); Zhernov et al. (2017), among others, yielded similar findings. In the aggregate, time-of-addition studies revealed that commingling viral particles with humic substances before adding cells to the mixture prevented viral fusion to cell membrane receptors; however, pre-treating cells with humic substances failed to influence the capacity of viral particles to adsorb to their target cell membrane receptors. Stated another way, (1) cells treated with humic substances before and after viral infection were found to be susceptible to viral adsorption; however, (2) cells exposed to humic substances at the time of infection do not experience the binding of viral particles to their cell surface membrane receptors, though viral entry is not otherwise inhibited; and (3) viral particles incubated with humic substances in advance of infection are not infectious due to viral inactivation and interference with the mechanics of viral attachment.

**TABLE 6:** In the Time of Addition study presented, humic acid or the reference compound, ribavirin, is added to virus-infected cells at discrete time points. Time point “0” represents prior to infection. The most effective antiviral effect is observed when cells were pretreated with humic acid. Though there was a decrease in the antiviral effect for humic acid between 1 h and 24 h relative to time “0,” ribavirin lost all activity by 24 h whereas humic acid remained active. Since the time from viral adsorption to the shedding of new influenza virus *in vitro* can begin after 6 h (World Health Organization, 2022), the IC_50_ data for humic acid at 24 h suggests that humic acid also limits new virus adsorption after a cycle of viral shedding. Given that cells *in vitro* were continuously exposed to humic acid material, this reasoned explanation is most likely.

Effect of time of addition on efficacy of humic acid and ribaviral reference compound against influenza virus type a (NIH, 2002) (New Caledonia/20/99M, H1N1, in MDCK cells)		
Time of addition, h	IC_50,_ mcg/ml Visual—Neutral red (NR) method	
	Humic acid	Ribavirin
0	5.5–5.5	7.5–6
1	14–15	6–5.5
2	16–17	7–8
4	10–10	7–7
8	14–14	9–12
24	48–55	>100–> 100

## Secondary mechanisms of action—Intracellular

By definition, humic acid is an opportunistic anti-viral molecule that inhibits viral adsorption to cell membrane receptors (1) during primary infection and (2) downstream of the replication cycle during viral shedding. Though the primary nexus for humic acid as an antiviral is extracellular, discreet observations have been reported for multiple intracellular activities. In studies evaluating Hepatits B virus, Pant et al. (2016) observed that humic acid (1) induces apoptosis of hepatic cancer cells *via* upregulation of caspase; (2) inhibits HBV-induced cell proliferation and autophagy *via* inhibition of HBx protein expression; (3) inhibits HBV DNA and HBsAg, and (4) inhibits HBV-induced autophagosome formation (Pant et al., 2016). The influence on cell cycle activities identified by Pant contrasts with mechanistic studies completed by Zhernov (2018) that further elaborated upon the influence of humic acid on viral replication. More specifically, Zhernov discovered that humic acid inhibits reverse transcriptase in an HIV-1 replication model, but not integrase. In 2002,Lu et alia, reported on the capacity of synthetic humate to disrupt viral RNA polymerase activity.

In 2000, Lüscher-Mattli noted that in their capacity as antiviral agents, anionic polymers such as humic acid inhibit syncytium formation between HIV-infected and normal CD4 T lymphocytes, which mirrored Meerbach’s parallel observation for a selection of 12 synthetic phenolic polymers (polyhydroxycarboxylates) (Meerbach et al., 2001; Lüscher-Mattli, 2000). Given findings that SARS-CoV-2 drives the formation of respiratory syncytia in those with severe respiratory disease (Lin et al., 2021), the potential of humic acid to limit the evolution of respiratory syncytia *in vivo* may be the subject of further study.

In unrelated assessments, Smirnova (2012) and Krezel (2016) conclude that humic acid behaves as an ionophore to facilitate the intracellular transport of zinc ions, which te Velthuis et al. (2010) showed have noteworthy antiviral effects against Sudden Acute Respiratory Syndrome (SARS), a clinical disease which some individuals developed during the 2003 coronavirus outbreak (Smirnova et al., 2012; Krężel and Maret, 2016; te Velthuis et al., 2010). Contemporary work regarding the antiviral influence of zinc on SARS-CoV-2 is substantial (Marreiro et al., 2021). Less well known is humic acid’s ability to stabilize zinc as well as selenium ions in chelate form, which enhances each ion’s bioavailability and anti-viral effect (Constantinescu-Aruxandei et al., 2018). Humic substances indirect protection of host systems from viral infection *via* the suppression of tumor necrosis factor alpha (TNF-α), prostaglandin E2 (PGE_2_) and cyclooxygenase 2 (COX-2) expression in human monocyte culture is also potentially clinically relevant (Hafez et al., 2020); as is its direct reduction of oxidative stress by complexing with intermediate free radicals and activation of the immune system *via* the promotion of IL-2 secretion (Vetvicka et al., 2013).

## Clinical humeomics: Translating humic acid into clinical practice

Humic substances have numerous potential clinical applications; however, the translation of what is known *in vitro* into clinical practice has been slow to mature. In the wake of SARS-CoV-2, the need for adjuvant antiviral therapies that are oral, deployable, and indifferent to viral antigenic shift is relevant. Translating the *in vitro* antiviral characteristics of humic acid into clinical practice would add to the clinician’s toolbox and the dialog within public health circles.

Pre-clinical studies have established two inflection points with humic substances. The first is that humic acid’s antiviral potential supersedes any contribution from fulvic acid, which in turn dwarfs the antiviral activity of shilajit. The second is that the translation of what is known regarding humic acid’s antiviral portfolio from pre-clinical studies to clinical application has been marred by concerns about efficacy, safety and tolerability.

**FIGURE 3:**
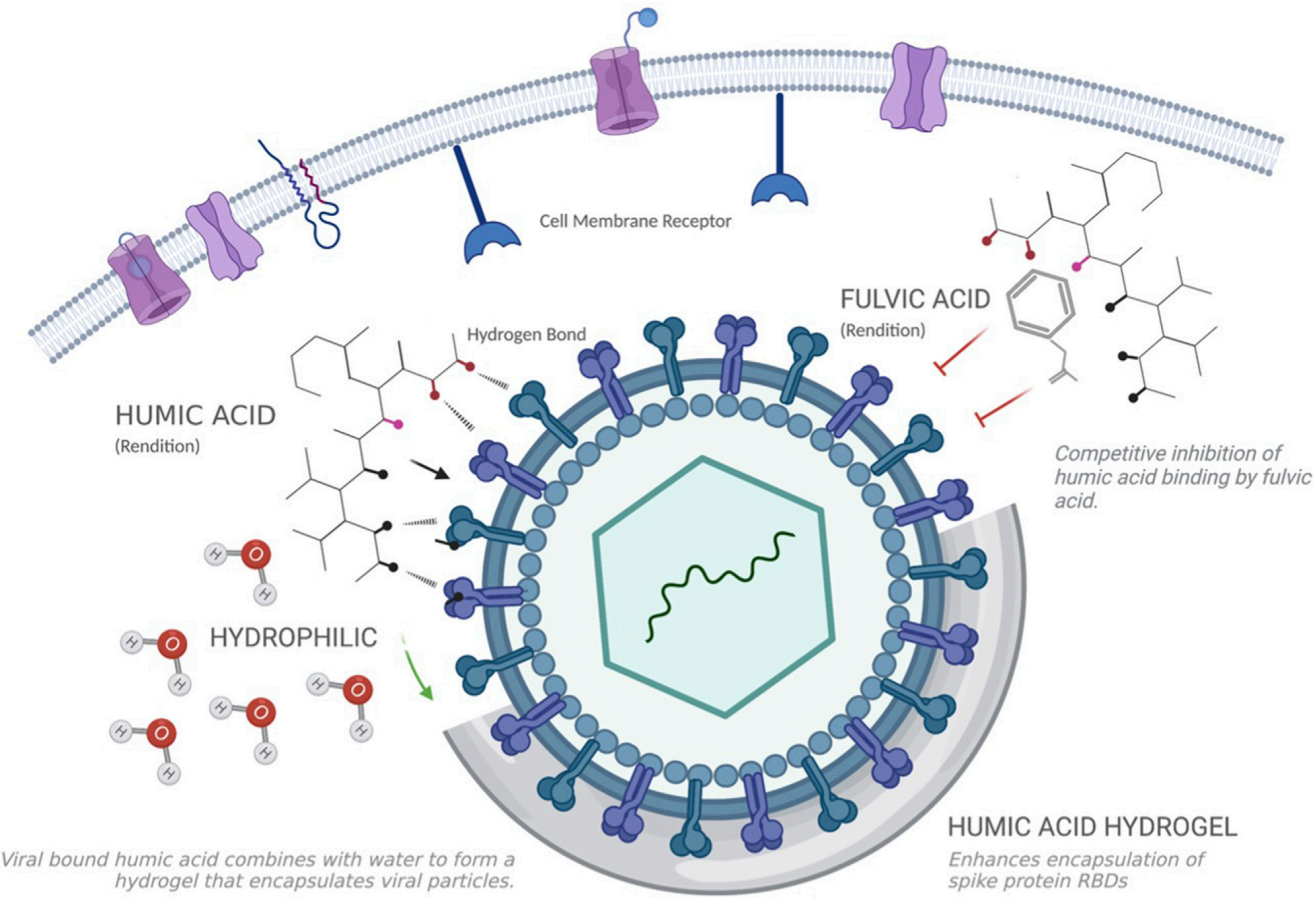
Humic acid binds to viral spike protein receptor binding domains (RBD) and inhibits viral fusion with target cell membrane receptors. The hydrophilic properties of the humic acid molecule attract water to form a hydrogel which encapsulates spike protein RBD and suspends the viral lifecycle. In the presence of fulvic acid, humic acid’s potential to bind spike protein RBDs is impaired, which is the molecule’s primary mechanism of action. Created with Biorender.com.

Regardless of the working group, pre-clinical studies that assessed the antiviral potential of humic acid relied on a pure extract of humic polyanions that is not generally available. In the United States, humic products historically available to the consumer are 30% humic acid, with limited exception. To rival the antiviral efficacy of what has been measured *in vitro*, humic acid needs to be isolated from its sister molecule, fulvic acid. In the absence of this purity, fulvic acid competitively inhibits the binding sites of the humic acid polyanion, rendering it less effective as an antiviral (Figure 3).

Subsequent to this clinical review, clinical experience with a purified and further enhanced iteration of humic acid that has been used to mitigate SARS-CoV-2 infection will be presented in tandem with data regarding significant TNF-α suppression, CD4^+^ cell population augmentation, and outcome enhancements in a clinical model of influenza infection. Prior concerns that humic acid has low bioavailability and a short plasma half-life will be reconciled against favorable clinical outcomes. In addition, an argument in favor of using humic acid as a prophylactic antiviral will be postulated given the opportunistic antiviral mechanics of the humic acid molecule, with guidance from Zhernov et alia's 2017 observation that humic acid loses 50% of its efficacy relative to fusion inhibition 7–8 h after infection (which mirrors the findings for the anti-HIV medication, AZT, at 7.7 +/− 0.2 h).

The clinical data to be presented further intends to balance clinical experience with humic acid’s potential disadvantages as a source of heavy metal toxicity, mineral chelation, and pro-coagulant potential. The data yields that individuals using 500 mg of a purified and further enhanced humic acid iteration for a minimum of 6 months exhibit no abnormalities of serum lead levels or clinically abnormal levels of calcium, iron, magnesium or zinc. Further, regardless of humic acid’s potential influence on the coagulation cascade, specifically Factors IIa, VIIa and Xa *via* a serum protein-humic substance aggregate, unremarkable data on protime (PT) and partial thromboplastin time (PTT) will also be presented and contrasted with expectations based on thromboelastography studies (Klöcking et al., 2013; Hafez et al., 2020).

## Conclusion

Humic substances, including humic acid, have been the subject of scientific inquiry since the early 1930s (Erdtman, 1933; Waksman, 1936). As a molecular class that has potential clinical relevance, they exhibit antiviral, anti-inflammatory, anti-oxidant, anti-cancer and gut promoting properties, but to date our understanding of this cadre of molecules has been limited by definitions, technology and clinical inquiry. The emergence of new technologies in the early 2000s reframed the concept of humic acid as a supramolecule rather than a molecular polymer. Numerous *in vitro* studies have chronicled the broad-spectrum antiviral capacity of humic substances against enveloped RNA and DNA viruses while also acknowledging their relative indifference to viral antigenic shift, among other features. These same studies established that humic substances, or polyanionic carboxylates, inhibit viral fusion to target cell membrane receptors as their primary mode of action, though other intracellular and broader indirect influence on the immune system and host viral response have also been reported. This being said, the translation of this body of *in vitro* research into clinical practice has been underwhelming based on concerns about bioavailability, a short plasma half-life and side effects. Future presentations will share insight regarding the successful deployment of humic acid as an antiviral in clinical practice and the basis for the pivot from pre-clinical studies into clinical practice.
